# Perinatal risk factors for premature ischaemic heart disease in a Swedish national cohort

**DOI:** 10.1136/bmjopen-2014-007308

**Published:** 2015-06-02

**Authors:** Bengt Zöller, Jan Sundquist, Kristina Sundquist, Casey Crump

**Affiliations:** 1 Center for Primary Health Care Research, Lund University, Malmö, Sweden; 2 Stanford Prevention Research Center, Stanford University School of Medicine, Stanford, California, USA; 3 Department of Medicine, Stanford University School of Medicine, Stanford, California, USA

**Keywords:** EPIDEMIOLOGY, PAEDIATRICS

## Abstract

**Objective:**

Several studies have reported associations between restricted fetal development, as shown by birth weight or birth length, and later ischaemic heart disease (IHD). However, few studies have examined the importance of these perinatal factors when taking into account gestational age at birth, hereditary factors, sociodemographic factors and comorbidities. This study investigated the importance of perinatal risk factors for premature IHD and myocardial infarction (MI) in a large Swedish cohort.

**Setting and participants:**

National cohort study of 1 970 869 individuals who were live-born in Sweden in 1973 through 1992, and followed up to 2010 (ages 18–38 years).

**Primary and secondary outcome measures:**

The main outcome was IHD, and the secondary outcome was MI.

**Results:**

A total of 668 individuals were diagnosed with IHD in 18.8 million person-years of follow-up. After adjusting for gestational age at birth, sociodemographic factors, comorbidities and family history of IHD, low fetal growth was associated with increased risk of IHD (HR for <−2 vs −1 to <1 SD, 1.54; 95% CI 1.15 to 2.07; p=0.004) and increased risk of MI (HR for <−2 vs −1 to <1 SD, 2.48; 95% CI 1.66 to 3.71; p<0.001) in young adulthood. In contrast, gestational age at birth was not associated with the risk of IHD or MI.

**Conclusions:**

In this large national cohort, low fetal growth was strongly associated with IHD and MI in young adulthood, independently of gestational age at birth, sociodemographic factors, comorbidities and family history of IHD.

**Strengths and limitations of this study**

- This birth cohort study is the largest nationwide study to examine the associations between restricted fetal development, as shown by birth weight, birth length or fetal growth and later ischaemic heart disease (IHD) or myocardial infarction (MI).
- Few studies have examined the importance of these perinatal factors when taking into account gestational age at birth, hereditary factors, sociodemographic factors and comorbidities.
- It highlights the importance of low fetal growth as a predictor for premature IHD and MI, independently of gestational age at birth, sociodemographic factors, comorbidities and family history of IHD.
- Study limitations include the unavailability of information on blood pressure, cholesterol, body mass index and smoking history. However, data were adjusted for parental socioeconomic factors that are related to life-style factors. Results were also adjusted for diagnosis of hypertension, diabetes, and cardiovascular and chromosomal anomalies.
- IHD and MI were identified from inpatient and outpatient diagnoses, thus we were unable to identify previously silent and undiagnosed events.

## Introduction

Low birth weight has been associated with increased risk of ischaemic heart disease (IHD) in adult life in a large number of studies.^1–18^ A fetal origin hypothesis by Barker postulates that fetal undernutrition in middle to late gestation results in altered gene expression, which predisposes for IHD in adult life.^19^ However, few studies of birth weight in relation to IHD have examined the specific contributions of fetal growth and gestational age at birth.^20–22^ Kaijser *et al*^21^ studied 6425 Swedish individuals born between 1925 and 1949, and concluded that low gestational age adjusted for birth weight was not a risk factor, whereas low birth weight adjusted for gestational age was an independent risk factor for IHD. In a recent birth cohort from Sweden, between 1983 and 1995, low gestational age and low birth weight were associated with cerebrovascular disease but not with IHD.^22^ Moreover, in a population-based study of men born in 1913, no association between birth weight and IHD or cardiovascular disease mortality was observed.^23^ Thus, conflicting results exist regarding the contribution of birth weight and gestational age to the risk of IHD.

Several mediators of the association between low birth weight and IHD have been suggested. Low birth weight has been associated with IHD risk factors such as obesity, hypertension and diabetes in adult life.^24^ ^25^ Preterm birth also has been independently associated with diabetes and hypertension in adult life.^26^ ^27^ Studies among Swedish twins have shown an association between perinatal factors and myocardial infarction (MI) among dizygotic but not monozygotic twins, suggesting that genetic and early environmental factors that operate independently of birth weight may be important.^28^ Frankel *et al*^3^ found that childhood or adult socioeconomic factors and other adult risk factors did not explain the association between low birth weight and IHD. This is consistent with a systematic review that found that adjusting for childhood or adult socioeconomic factors did not alter the overall age-adjusted and sex-adjusted association between low birth weight and risk of IHD.^20^ However, few studies have taken into account hereditary factors, that is, family history of IHD, when examining the association between birth weight and IHD.^22^ Most studies have examined only IHD mortality and not IHD or MI morbidity. In addition, most previous studies have examined cohorts that were born before 1950.^1–18^ Because perinatal care has improved over time, it is unclear to what extent earlier findings are generalisable to more recent birth cohorts.

We conducted a national cohort study in Sweden, to examine the association between perinatal factors and premature IHD and MI. A national cohort of infants born from 1973 through 1992 was followed up until 2010 for IHD and MI. We tested whether birth weight, birth length, standardised fetal growth or gestational age at birth is associated with an increased risk of IHD or MI independently of hereditary factors, sociodemographic factors and comorbidities.

## Methods

### Study population

We identified 1 984 858 individuals in the Swedish Birth Registry who were live-born from 1973 through 1992 and still living in Sweden at age 18 years. We excluded 4625 (0.2%) persons who had missing information for birth weight, and 6069 (0.3%) others who had missing information for gestational age at birth. To remove possible coding errors, we excluded 3061 (0.2%) who had a reported birth weight more than 4 SDs above or below the mean birth weight for gestational age and sex, based on a Swedish reference growth curve.^29^ To examine the risk of incident IHD and MI in young adulthood, we excluded 234 (0.01%) others with a prior diagnosis of IHD before age 18 years. A total of 1 970 869 individuals (99.3% of the original cohort) remained for inclusion in the study.

### IHD ascertainment

The study cohort was followed up for the earliest incidence of IHD from age 18 years through 31 December 2010 (maximum attained age was 38 years). IHD was identified using primary and secondary diagnoses from the International Classification of Diseases (ICD), revisions 9 and 10 (codes 410–414 in ICD-9 and I20-I25 in ICD-10) in the Swedish Hospital Registry, Outpatient Registry and/or Cause of Death Registry. The Swedish Hospital Registry contains all primary and secondary hospital discharge diagnoses for six populous counties in southern Sweden, starting in 1964, and with nationwide coverage since 1987; and the Swedish Outpatient Registry contains all outpatient specialty clinic diagnoses nationwide starting in 2001. The Cause of Death Registry includes all deaths nationwide since 1964 for all persons registered in Sweden at the time of death. In secondary analyses, acute MI (MI; codes 410 in ICD-9 and I21 in ICD-10) was examined separately. The validity of IHD or MI diagnosis in the Hospital Registry has been reported to be ∼95%.^30–32^

### Perinatal, familial and comorbidity variables

Perinatal and familial characteristics that may be associated with IHD were identified from the Swedish Birth Registry and national census data, which were linked using an anonymous personal identification number.^33^ ^34^ The following variables were examined as predictors of interest or adjustment variables: age (adjusted for as the Cox regression model time scale); sex (male or female); gestational age at birth (based primarily on maternal report of last menstrual period in the 1970s, at which time ultrasound estimation was gradually introduced until it was used exclusively starting in the 1990s, modelled alternatively as a categorical (<37, 37–41, >42 weeks) or continuous variable); fetal growth (a standardised variable defined as the number of SD from the mean birth weight for gestational age and sex based on a Swedish reference growth curve,^29^ modelled alternatively as a categorical (<−2; −2 to <−1; −1 to <1; >1 SD) or continuous variable); birth weight (modelled alternatively as a categorical (<2500, 2500–3999, >4000 g) or continuous variable); birth length (crown-heel length in cm, modelled alternatively as a categorical (<48, 48–52, >53 cm) or continuous variable); multiple birth (singleton versus twin or higher order); birth order (1, 2, >3); maternal age at birth (<20, 20–24, 25–29, 30–34, >35 years); maternal marital status (married/cohabiting, never married, divorced/widowed); maternal and paternal education level (compulsory high school or less (<9 years), practical high school or some theoretical high school (10–11 years), theoretical high school and/or some college (12–14 years), college and/or postgraduate study (>15 years); examined separately for mothers and fathers); any inpatient or outpatient diagnosis of cardiovascular and chromosomal anomalies or syndromes (eg, including Marfan syndrome) (codes 746–747 and 759 in ICD-8, 746–747, 758 and 759.8 in ICD-9, and Q20–Q28, Q87 and Q90–Q99 in ICD-10), diabetes mellitus (codes 250 in ICD-9 and E10–E14 in ICD-10), or hypertension (codes 401–405 in ICD-9 and I10–I15 in ICD-10); and parental history of IHD (yes or no; identified from the Swedish Hospital Registry in 1964–2010 and Outpatient Registry in 2001–2010 (codes 420 in ICD-7, 410–414 in ICD-8/9 and I20–I25 in ICD-10), not self-reported, thus enabling unbiased ascertainment during the study period). Chronic obstructive pulmonary disease (codes 491–492 in ICD-9 and J41–J44 in ICD-10) was also examined but, there were only five cases among persons with IHD, which were too few for further analysis as a predictor or adjustment variable.

### Statistical analysis

Cox proportional hazards regression was used to estimate HRs and 95% CIs for associations between perinatal or familial variables, and IHD. Individuals were censored at the time of death from any cause other than IHD (n=7158; 0.4%), or at the time of emigration as determined by the absence of a Swedish residential address in census data (n=38 440; 2.0%). Two different adjusted models were used: The first was adjusted for age (as the model time scale) and sex, and the second was further adjusted for other variables that were found to be associated with IHD (fetal growth, gestational age at birth, multiple birth, maternal marital status, maternal and paternal education, cardiovascular and chromosomal anomalies or syndromes, diabetes, hypertension and parental history of IHD). First-order interactions between sex and other variables were examined using likelihood ratio tests. The proportional hazards assumption was evaluated by graphical assessment of log-log plots and was met in each of the models. All statistical tests were two sided and used an α-level of 0.05. All analyses were conducted using Stata statistical software, V.12.1.

## Results

Among the 1 970 869 persons in this cohort, 668 (0.03%) IHD cases were identified in 18.8 million person-years of follow-up (35.5 cases per million person-years). The median age at IHD diagnosis was 23.5 years (mean 27.5, SD 5.3). Males had a higher risk of IHD than females (fully adjusted HR, 1.75; 95% CI 1.49 to 2.05; p<0.001) (table 1). However, the associations between other variables and IHD did not vary by sex (p>0.05 for each interaction, including for fetal growth (p=0.78), gestational age at birth (p=0.86) and birth weight (p=0.65)), hence non-stratified risk estimates are presented in table 1.

**Table 1:** HRs for associations between perinatal or familial factors and ischaemic heart disease (IHD) in young adulthood (ages 18–38 years)

	IHD (N=668)	No IHD (N=1 970 201)	Adjusted model 1*		Adjusted model 2†	
			HR (95% CI)	p Value	HR (95% CI)	p Value
Sex						
Male	435 (65.1)	1 011 914 (51.4)	1.76 (1.50 to 2.07)	<0.001	1.75 (1.49 to 2.05)	<0.001
Female	233 (34.9)	958 287 (48.6)	1.00		1.00	
Fetal growth (SD)						
<−2	54 (8.1)	73 191 (3.7)	1.89 (1.42 to 2.51)	<0.001	1.54 (1.15 to 2.07)	0.004
−2 to <−1	137 (20.5)	318 840 (16.2)	1.25 (1.03 to 1.52)	0.02	1.16 (0.95 to 1.41)	0.14
−1 to <1	400 (59.9)	1 302 754 (66.1)	1.00		1.00	
>1	77 (11.5)	275 416 (14.0)	0.96 (0.76 to 1.23)	0.77	0.98 (0.77 to 1.26)	0.90
Per additional 1 SD (trend test)			0.88 (0.82 to 0.95)	<0.001	0.93 (0.87 to 0.99)	0.04
Gestational age at birth (weeks)						
<37	44 (6.6)	105 352 (5.3)	1.36 (1.00 to 1.85)	0.05	1.07 (0.78 to 1.47)	0.68
37–41	545 (81.6)	1 680 326 (85.3)	1.00		1.00	
>42	79 (11.8)	184 523 (9.4)	1.03 (0.82 to 1.31)	0.79	0.97 (0.77 to 1.24)	0.83
Per additional 1 week (trend test)			0.98 (0.94 to 1.02)	0.26	1.00 (0.96 to 1.05)	0.84
Birth weight (g)						
<2500	40 (6.0)	76 055 (3.9)	1.70 (1.23 to 2.34)	0.001	1.32 (0.90 to 1.95)	0.16
2500–3999	520 (77.8)	1 557 606 (79.1)	1.00		1.00	
>4000	108 (16.2)	336 540 (17.1)	0.94 (0.76 to 1.15)	0.54	0.98 (0.80 to 1.21)	0.88
Per 1000 g (trend test)			0.77 (0.67 to 0.88)	<0.001	0.84 (0.73 to 0.96)	0.009
Birth length (cm)						
<48	84 (12.6)	194 563 (9.9)	1.44 (1.14 to 1.81)	0.002	1.17 (0.90 to 1.52)	0.24
48–52	492 (73.7)	1 452 416 (73.7)	1.00		1.00	
>53	91 (13.6)	311 550 (15.8)	0.75 (0.60 to 0.94)	0.01	0.77 (0.62 to 0.97)	0.03
Unknown	1 (0.1)	11 672 (0.6)	0.48 (0.07 to 3.45)	0.47	0.41 (0.06 to 2.98)	0.38
Per cm (trend test)			0.94 (0.91 to 0.97)	<0.001	0.96 (0.93 to 1.00)	0.04
Multiple birth status						
Singleton	647 (96.9)	1 932 944 (98.1)	1.00		1.00	
Twin or higher order	21 (3.1)	37 257 (1.9)	1.92 (1.25 to 2.97)	0.003	1.63 (1.04 to 2.57)	0.03
Birth order						
1	275 (41.2)	824 592 (41.9)	1.00		1.00	
2	250 (37.4)	717 996 (36.4)	1.05 (0.89 to 1.25)	0.57	1.03 (0.87 to 1.23)	0.72
>3	143 (21.4)	427 613 (21.7)	1.13 (0.92 to 1.38)	0.24	0.97 (0.78 to 1.21)	0.81
Per 1 higher birth order (trend test)			1.06 (0.96 to 1.17)	0.24	0.99 (0.89 to 1.10)	0.88
Maternal age at delivery (years)						
<20	41 (6.1)	60 493 (3.1)	1.46 (1.05 to 2.02)	0.03	1.21 (0.85 to 1.72)	0.28
20–24	184 (27.5)	463 194 (23.5)	1.03 (0.86 to 1.25)	0.72	0.96 (0.79 to 1.17)	0.67
25–29	258 (38.6)	739 852 (37.5)	1.00		1.00	
30–34	129 (19.3)	491 675 (25.0)	0.85 (0.69 to 1.05)	0.14	0.82 (0.67 to 1.02)	0.08
>35	56 (8.4)	214 987 (10.9)	0.98 (0.74 to 1.31)	0.92	0.85 (0.64 to 1.14)	0.29
Per each higher category (trend test)			0.92 (0.85 to 0.99)	0.03	0.93 (0.86 to 1.01)	0.08
Maternal marital status						
Married/cohabiting	416 (62.3)	1 460 600 (74.1)	1.00		1.00	
Never married	170 (25.4)	297 723 (15.1)	1.08 (0.90 to 1.29)	0.41	0.97 (0.81 to 1.17)	0.78
Divorced/widowed	82 (12.3)	211 878 (10.8)	1.33 (1.05 to 1.69)	0.02	1.25 (0.98 to 1.59)	0.07
Maternal education (years)						
<9	226 (33.8)	468 879 (23.8)	1.00		1.00	
10–11	254 (38.0)	759 323 (38.5)	0.89 (0.75 to 1.07)	0.22	0.96 (0.80 to 1.15)	0.67
12–14	92 (13.8)	445 653 (22.6)	0.60 (0.47 to 0.77)	<0.001	0.72 (0.56 to 0.93)	0.01
>15	48 (7.2)	214 015 (10.9)	0.55 (0.41 to 0.76)	<0.001	0.75 (0.53 to 1.06)	0.10
Unknown	48 (7.2)	82 331 (4.2)	1.38 (1.01 to 1.88)	0.04	1.32 (0.96 to 1.82)	0.09
Per each higher category (trend test)			0.80 (0.74 to 0.88)	<0.001	0.88 (0.80 to 0.97)	0.01
Paternal education (years)						
<9	246 (36.8)	538 836 (27.3)	1.00		1.00	
10–11	203 (30.4)	631 893 (32.1)	0.97 (0.80 to 1.17)	0.73	1.01 (0.84 to 1.22)	0.93
12–14	117 (17.5)	437 434 (22.2)	0.68 (0.55 to 0.85)	0.001	0.79 (0.63 to 0.99)	0.05
>15	53 (7.9)	260 395 (13.2)	0.52 (0.39 to 0.71)	<0.001	0.69 (0.50 to 0.97)	0.03
Unknown	49 (7.3)	101 643 (5.2)	1.21 (0.89 to 1.65)	0.22	1.14 (0.83 to 1.57)	0.41
Per each higher category (trend test)			0.82 (0.75 to 0.88)	<0.001	0.90 (0.82 to 0.98)	0.02
Cardiovascular and chromosomal anomalies or syndromes						
No	613 (91.8)	1 954 894 (99.2)	1.00		1.00	
Yes	55 (8.2)	15 307 (0.8)	13.68 (10.38 to 18.03)	<0.001	10.30 (7.77 to 13.66)	<0.001
Diabetes						
No	606 (90.7)	1 953 382 (99.1)	1.00		1.00	
Yes	62 (9.3)	16 819 (0.9)	10.49 (8.08 to 13.63)	<0.001	5.56 (4.15 to 7.44)	<0.001
Hypertension						
No	604 (90.4)	1 964 560 (99.7)	1.00		1.00	
Yes	64 (9.6)	5641 (0.3)	23.37 (18.05 to 30.25)	<0.001	10.60 (7.91 to 14.20)	<0.001
Parental history of IHD						
No	514 (76.9)	1 795 779 (91.1)	1.00		1.00	
Yes	154 (23.1)	174 422 (8.9)	2.07 (1.73 to 2.48)	<0.001	1.85 (1.54 to 2.22)	<0.001

*Adjusted for age and sex.

†Adjusted for age, sex, fetal growth, gestational age at birth, multiple birth, maternal marital status, maternal and paternal education, cardiovascular and chromosomal anomalies or syndromes, diabetes, hypertension and parental history of IHD.

Birth weight and birth length were each examined in separate models as alternatives to the standardised fetal growth variable. The reference category for all variables is indicated by an HR of 1.00. Missing data were excluded for trend tests.

### IHD results

Fetal growth was inversely associated with the risk of IHD in young adulthood (table 1). Adjusting for age and sex, lowest fetal growth was associated with nearly twice the risk of IHD relative to normal fetal growth (HR for <−2 SD vs −1 to <1 SD, 1.89; 95% CI 1.42 to 2.51; p<0.001). Further adjustment for gestational age at birth, sociodemographic factors, comorbidities and parental history of IHD resulted in a moderately attenuated risk estimate that remained highly significant (fully adjusted HR for <−2 SD vs −1 to <1 SD, 1.54; 95% CI 1.15 to 2.07; p=0.004), and an inverse trend remained across the full range of fetal growth (fully adjusted HR per additional 1 SD, 0.93; 95% CI 0.87 to 0.99; p=0.04) (table 1, adjusted model 2). A similar inverse association was also found between birth weight or birth length and IHD (table 1). In contrast, gestational age at birth was not associated with the risk of IHD in young adulthood (table 1).

Other strong independent risk factors for IHD in this cohort included hypertension, diabetes, cardiovascular and chromosomal anomalies or syndromes, parental history of IHD, multiple birth and low maternal or paternal education (table 1). Birth order, maternal age and maternal marital status were not significantly associated with IHD.

### MI results

The findings for MI were overall similar to those for IHD but stronger in magnitude (table 2). Low fetal growth was strongly associated with increased risk of MI, and the risk estimates were only modestly affected by adjustment for gestational age at birth, sociodemographics, comorbidities and family history. In the fully adjusted model, lowest fetal growth was associated with more than twice the risk of MI relative to normal fetal growth (HR for <−2 SD vs −1 to <1 SD, 2.48; 95% CI 1.66 to 3.71; p<0.001), and a highly significant inverse trend was found across the full range of fetal growth (HR per additional 1 SD, 0.78; 95% CI 0.69 to 0.87; p<0.001) (table 2, adjusted model 2). A similar, strong inverse association was also found between birth weight or birth length and MI (table 2). In contrast, gestational age at birth was not associated with the risk of MI in young adulthood (table 2).

**Table 2:** HRs for associations between perinatal or familial factors and acute MI in young adulthood (ages 18–38 years)

	MI (N=239)	No MI (N=1 970 630)	Adjusted model 1*		Adjusted model 2†	
			HR (95% CI)	p Value	HR (95% CI)	p Value
Sex						
Male	170 (71.1)	1 012 179 (51.4)	2.32 (1.76 to 3.07)	<0.001	2.33 (1.76 to 3.08)	<0.001
Female	69 (28.9)	958 451 (48.6)	1.00		1.00	
Fetal growth (SD)						
<−2	32 (13.4)	73 213 (3.7)	2.36 (1.78 to 3.12)	<0.001	2.48 (1.66 to 3.71)	<0.001
−2 to <−1	53 (22.2)	318 924 (16.2)	1.45 (1.05 to 1.99)	0.02	1.31 (0.95 to 1.80)	0.10
−1 to <1	132 (55.2)	1 303 022 (66.1)	1.00		1.00	
>1	22 (9.2)	275 471 (14.0)	0.84 (0.53 to 1.32)	0.45	0.86 (0.54 to 1.34)	0.50
Per additional 1 SD (trend test)			0.71 (0.63 to 0.80)	<0.001	0.78 (0.69 to 0.87)	<0.001
Gestational age at birth weeks)						
<37	16 (6.7)	105 380 (5.3)	1.44 (0.86 to 2.39)	0.17	1.05 (0.62 to 1.78)	0.85
37–41	188 (78.7)	1 680 683 (85.3)	1.00		1.00	
>42	35 (14.6)	184 567 (9.4)	1.29 (0.90 to 1.84)	0.17	1.12 (0.77 to 1.62)	0.55
Per additional 1 week (trend test)			1.00 (0.93 to 1.07)	0.94	1.02 (0.96 to 1.10)	0.49
Birth weight (g)						
<2500	22 (9.2)	76 073 (3.9)	2.71 (1.74 to 4.22)	<0.001	2.26 (1.31 to 3.91)	0.004
2500–3999	182 (76.2)	1 557 944 (79.1)	1.00		1.00	
>4000	35 (14.6)	336 613 (17.1)	0.85 (0.59 to 1.22)	0.37	0.86 (0.59 to 1.24)	0.42
Per 1000 g (trend test)			0.58 (0.46 to 0.71)	<0.001	0.61 (0.47 to 0.79)	<0.001
Birth length (cm)						
<48	36 (15.1)	194 611 (9.9)	1.78 (1.24 to 2.55)	0.002	1.43 (0.95 to 2.15)	0.09
48–52	176 (73.6)	1 452 732 (73.7)	1.00		1.00	
>53	27 (11.3)	311 614 (15.8)	0.59 (0.39 to 0.89)	0.01	0.59 (0.39 to 0.89)	0.01
Unknown	0 (0.0)	11 673 (0.6)	NE	NE	NE	NE
Per cm (trend test)			0.91 (0.87 to 0.95)	<0.001	0.93 (0.88 to 0.98)	0.006
Multiple birth status						
Singleton	228 (95.4)	1 933 363 (98.1)	1.00		1.00	
Twin or higher order	11 (4.6)	37 267 (1.9)	2.91 (1.59 to 5.34)	0.001	2.25 (1.19 to 4.25)	0.01
Birth order						
1	104 (43.5)	824 763 (41.9)	1.00		1.00	
2	81 (33.9)	718 165 (36.4)	0.90 (0.68 to 1.21)	0.49	0.90 (0.67 to 1.22)	0.50
>3	54 (22.6)	427 702 (21.7)	1.15 (0.83 to 1.60)	0.40	0.95 (0.66 to 1.35)	0.76
Per 1 higher birth order (trend test)			1.05 (0.89 to 1.24)	0.56	0.96 (0.81 to 1.15)	0.69
Maternal age at delivery (years)						
<20	15 (6.3)	60 519 (3.1)	1.53 (0.89 to 2.66)	0.13	1.19 (0.66 to 2.13)	0.57
20–24	79 (33.1)	463 299 (23.5)	1.31 (0.97 to 1.78)	0.08	1.17 (0.85 to 1.61)	0.33
25–29	86 (36.0)	740 024 (37.6)	1.00		1.00	
30–34	41 (17.1)	491 763 (25.0)	0.83 (0.57 to 1.21)	0.33	0.80 (0.55 to 1.17)	0.25
>35	18 (7.5)	215 025 (10.9)	1.00 (0.60 to 1.67)	0.99	0.83 (0.50 to 1.39)	0.49
Per each higher category (trend test)			0.85 (0.75 to 0.97)	0.01	0.88 (0.76 to 1.00)	0.06
Maternal marital status						
Married/cohabiting	147 (61.5)	1 460 869 (74.1)	1.00		1.00	
Never married	67 (28.0)	297 826 (15.1)	1.12 (0.84 to 1.50)	0.43	0.95 (0.71 to 1.27)	0.72
Divorced/widowed	25 (10.5)	211 935 (10.8)	1.18 (0.77 to 1.81)	0.44	1.06 (0.69 to 1.63)	0.79
Maternal education (years)						
<9	89 (37.2)	469 016 (23.8)	1.00		1.00	
10–11	91 (38.1)	759 486 (38.5)	0.84 (0.63 to 1.13)	0.25	0.94 (0.70 to 1.26)	0.68
12–14	30 (12.6)	445 715 (22.6)	0.52 (0.34 to 0.79)	0.002	0.68 (0.44 to 1.05)	0.08
>15	10 (4.2)	214 053 (10.9)	0.30 (0.16 to 0.58)	<0.001	0.48 (0.23 to 0.97)	0.04
Unknown	19 (7.9)	82 360 (4.2)	1.45 (0.88 to 2.38)	0.15	1.34 (0.81 to 2.24)	0.26
Per each higher category (trend test)			0.71 (0.61 to 0.82)	<0.001	0.81 (0.69 to 0.96)	0.02
Paternal education (years)						
<9	99 (41.4)	538 983 (27.4)	1.00		1.00	
10–11	70 (29.3)	632 026 (32.1)	0.86 (0.64 to 1.17)	0.35	0.92 (0.67 to 1.25)	0.58
12–14	37 (15.5)	437 514 (22.2)	0.55 (0.37 to 0.80)	0.002	0.68 (0.46 to 1.00)	0.05
>15	13 (5.4)	260 435 (13.2)	0.33 (0.18 to 0.59)	<0.001	0.54 (0.29 to 1.02)	0.06
Unknown	20 (8.4)	101 672 (5.2)	1.28 (0.79 to 2.07)	0.32	1.214 (0.74 to 1.99)	0.45
Per each higher category (trend test)			0.72 (0.63 to 0.83)	<0.001	0.83 (0.71 to 0.97)	0.02
Cardiovascular and chromosomal anomalies or syndromes						
No	221 (92.5)	1 955 286 (99.2)	1.00		1.00	
Yes	18 (7.5)	15 344 (0.8)	12.75 (7.89 to 20.62)	<0.001	9.14 (5.60 to 14.90)	<0.001
Diabetes						
No	205 (85.8)	1 953 783 (99.1)	1.00		1.00	
Yes	34 (14.2)	16 847 (0.9)	16.55 (11.51 to 23.79)	<0.001	9.60 (6.35 to 14.50)	<0.001
Hypertension						
No	215 (90.0)	1 964 949 (99.7)	1.00		1.00	
Yes	24 (10.0)	5681 (0.3)	23.13 (15.16 to 35.30)	<0.001	7.55 (4.66 to 12.24)	<0.001
Parental history of IHD						
No	169 (70.7)	1 796 124 (91.1)	1.00		1.00	
Yes	70 (29.3)	174 506 (8.9)	2.73 (2.06 to 3.61)	<0.001	2.36 (1.78 to 3.13)	<0.001

*Adjusted for age and sex.

†Adjusted for age, sex, fetal growth, gestational age at birth, multiple birth, maternal and paternal education, cardiovascular and chromosomal anomalies or syndromes, diabetes, hypertension and parental history of IHD.

Birth weight and birth length were each examined in separate models as alternatives to the standardised fetal growth variable. The reference category for all variables is indicated by an HR of 1.00. Missing data were excluded for trend tests.

IHD, ischaemic heart disease; MI, myocardial infarction; NE, not estimable.

A sensitivity analysis that excluded persons with any congenital anomalies (n=104 473; 5.3%, codes 740–759 in ICD-8/9 and codes Q00–Q99 in ICD-10) produced very similar risk estimates as the main analysis, including those for fetal growth (IHD: fully adjusted HR per additional 1 SD, 0.92; 95% CI 0.85 to 0.99; p=0.03; MI: fully adjusted HR per additional 1 SD, 0.80; 95% CI 0.70 to 0.91; p=0.001).

## Discussion

In this large national cohort study, we found that low fetal growth was associated with increased risk of IHD and MI in young adulthood, independently of gestational age at birth, sociodemographic factors, comorbidities and family history of IHD. Thus, low fetal growth adds to traditional risk factors for premature IHD and MI.^35^ These findings confirm that low fetal growth is the component of low birth weight accounting for its previously observed association with IHD,^1–18^ and further show that this association holds true in a more recent birth cohort from a developed country. Moreover, we found that the association between restricted fetal growth and premature MI was stronger than for IHD. In contrast, gestational age at birth was not associated with IHD or MI risk, consistent with previous Swedish birth cohort studies from 1925 to 1949.^21^ ^22^ ^36^ The present findings are in contrast with a recent study of venous thromboembolism (VTE) that found a weak association with low fetal growth but a stronger association with preterm birth.^34^ Thus, the perinatal factors predisposing for IHD/MI appear to be partially different from those predisposing to VTE in adult life.^34^ Although VTE and IHD/MI are frequently comorbid, the present study adds to other evidence showing differences in their risk factors.^37^

The associations we observed between low fetal growth and IHD/MI were only partly explained by socioeconomic factors, hereditary factors and common comorbidities. The remaining associations are likely related to other unmeasured long-term complications of restricted fetal growth, although the specific underlying mechanisms are not well-established. One hypothesis is that fetal undernutrition or malnutrition leads to altered gene expression, resulting in physiological alterations such as insulin resistance, vascular endothelial dysfunction and abnormal neuroendocrine stress responses.^19^ ^38–40^ Unknown genetic factors may also be important.^28^ There is a negative correlation between maternal blood pressure during pregnancy and birth weight.^41^ Other possible confounding factors include maternal smoking^42^ and malformation syndromes,^43^ which may contribute to both small size at birth, and a possible increased risk of IHD and MI. Our findings were adjusted for cardiovascular and chromosomal anomalies, but information about maternal smoking was lacking. Though we adjusted for parental socioeconomic factors as a proxy for life-style factors such as smoking, the influence of parental smoking on the relationship between fetal growth and adult IHD or MI warrants further investigation.

The most important strength of the current study was its ability to examine the association between perinatal risk factors and risk of IHD/MI in young adulthood using nationwide birth, hospital, outpatient and death registry data for a large national cohort. The results were adjusted for other perinatal risk factors as well as other broadly measured potential confounders. Information on common comorbidities enabled us to examine their influence on the main findings.

Study limitations include the unavailability of information on blood pressure, cholesterol, body mass index and smoking history. However, the results were adjusted for parental socioeconomic factors, which are related to life-style factors such as smoking, and for diagnoses of hypertension, diabetes, and cardiovascular and chromosomal anomalies. IHD and MI were identified from inpatient and outpatient specialty clinic diagnoses, thus we were unable to identify previously silent and undiagnosed events. Information on genetic risk factors was unavailable, although the results were adjusted for family history of IHD. It is also unclear to what extent our findings will apply to later birth cohorts or older ages. However, generalisability to other populations seems likely, given the overall consistency of results across different birth cohorts.^1–18^

In summary, among individuals born in Sweden between 1973 and 1992, low fetal growth was a strong independent risk factor for IHD and MI in young adulthood. In contrast, gestational age at birth was not associated with the risk of IHD or MI. These findings call for better awareness of the long-term risk of IHD/MI among growth-restricted infants.
